# Chikungunya Outbreak, French Polynesia, 2014

**DOI:** 10.3201/eid2104.141741

**Published:** 2015-04

**Authors:** Maite Aubry, Anita Teissier, Claudine Roche, Vaea Richard, Aurore Shan Yan, Karen Zisou, Eline Rouault, Véronique Maria, Stéphane Lastère, Van-Mai Cao-Lormeau, Didier Musso

**Affiliations:** Institut Louis Malardé, Tahiti, French Polynesia (M. Aubry, A. Teissier, C. Roche, V. Richard, A. Shan Yan, K. Zisou, E. Rouault, V. Maria, V.-M. Cao-Lormeau, D. Musso);; Centre Hospitalier de la Polynésie française, Tahiti (S. Lastère)

**Keywords:** chikungunya, chikungunya virus, CHIKV, French Polynesia, Pacific, outbreak, arboviruses, viruses

**To the Editor:** Chikungunya virus (CHIKV), an arthropod-borne virus (arbovirus) of the family *Togaviridae*, genus *Alphavirus*, is transmitted by mosquitoes of the *Aedes* genus, especially *Ae. aegypti* and *Ae. albopictus* (*1*). The main clinical manifestations of CHIKV infections are sudden high fever, headache, back pain, myalgia, arthralgia affecting mainly the extremities, and rash.

CHIKV emerged in the Pacific region in New Caledonia in March 2011. Additional outbreaks occurred in Papua New Guinea in June 2012; Yap State (Federated States of Micronesia) in August 2013; Tonga in April 2014; and American Samoa, Samoa, and Tokelau in July 2014 (*2*). Phylogenetic analysis of CHIKV strains showed the existence of 3 lineages: West African, Asian, and East/Central/South African (*1*).

French Polynesia is a French territory in the South Pacific, with 270,000 inhabitants living on 5 archipelagoes. Arboviruses are a common cause of outbreaks in French Polynesia: the last dengue virus (DENV) outbreaks caused by DENV-1 and DENV-3 occurred in 2013 (*3*), and DENV-1 still circulates. French Polynesia also experienced the largest Zika virus (ZIKV) outbreak ever reported during October 2013–April 2014 (*4*). In May 2014, CHIKV infection was detected for the first time in French Polynesia in a traveler returning from Guadeloupe, (*5*) where a chikungunya outbreak was ongoing (*6*).

In late September 2014, an increasing number of patients with fever and rash who tested negative for DENV and ZIKV by real-time reverse transcription PCR (RT-PCR) were recorded by the French Polynesia Department of Health on the south coast of Tahiti, French Polynesia’s main island. Serum samples collected from 19 of these patients were tested for CHIKV by RT-PCR using previously reported primers and a probe (*7*). Seven of the 19 (37%) were positive; all 7 were autochthonous. The first specimen that tested positive for CHIKV had been collected from a patient on September 25, and by October 25, a total of 318 patients were confirmed by RT-PCR to be infected by CHIKV. Nearly all districts of Tahiti were affected, and cases were reported on 4 of French Polynesia’s 5 archipelagoes.

Partial sequencing of the CHIKV E1 gene of a strain isolated from a patient and collected on September 29 (strain PF14-290914-16, GenBank accession no. KM985619) was performed as previously reported (*8*). Phylogenetic analysis showed that French Polynesia’s CHIKV strain belongs to the Asian lineage and is more closely related to a strain collected in the British Virgin Islands in 2014 (VG14/99659) and to the French Polynesian strain imported from Guadeloupe in May 2014 (PF14-270514-51impGP), with 99.9% homology, than to the strains that recently circulated in Yap State (FM13/3807), Tonga (TO14-080414-3007 and TO14-080414-3042), and New Caledonia (NC11-568) (Figure).

**Figure:**
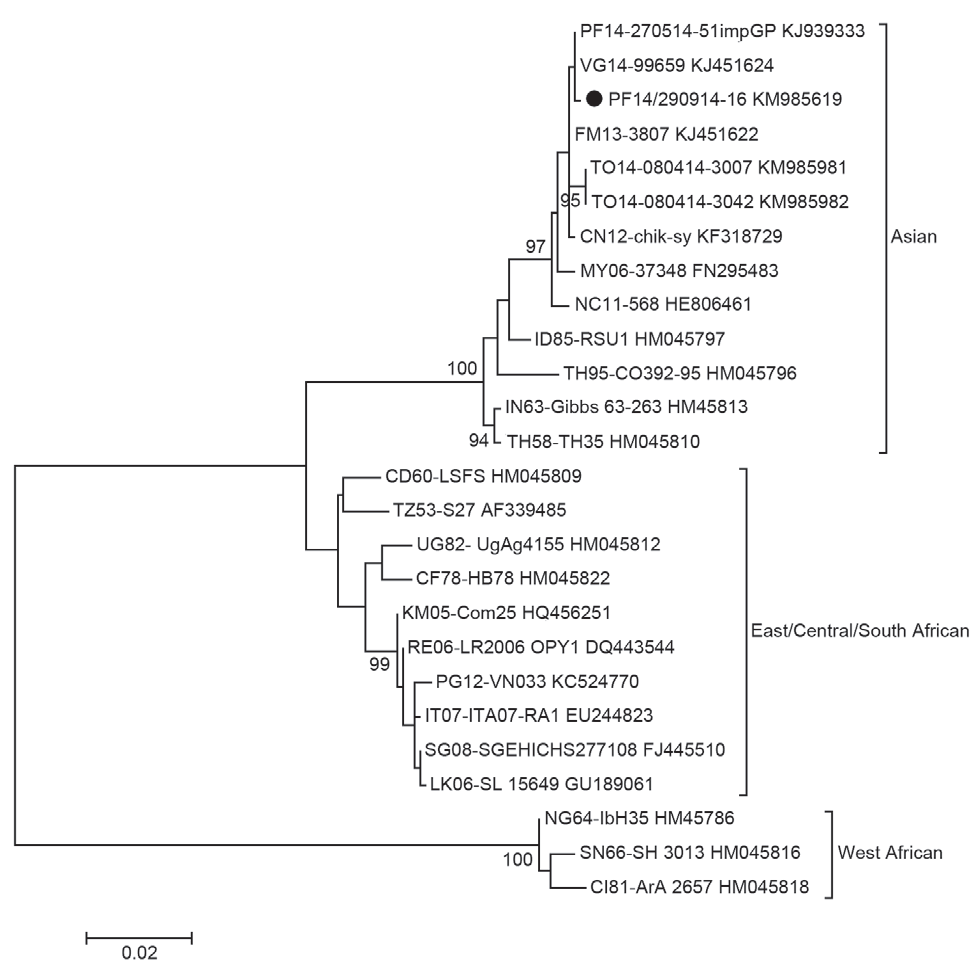
Phylogenetic analysis of chikungunya virus strain isolated in French Polynesia on September 29, 2014. The evolutionary history was inferred by using the maximum-likelihood method based on the Kimura 2-parameter model. The percentage of trees in which the associated taxa clustered together is shown for values >90 next to the branches (1,000 replicates). Evolutionary analyses were conducted in MEGA6 (http://www.megasoftware.net/mega.php). Each strain is labeled by country (iso country code, 2-letter) and date of origin/strain name/GenBank accession number. The chikungunya virus strain isolated in French Polynesia on September 2014 is marked with a black circle. Scale bar indicates nucleotide substitutions per site.

No cases of CHIKV infection were reported in French Polynesia within the 4 months after the imported case detected on May 25, 2014. Because of the active, ongoing circulation of CHIKV in the Pacific, introduction of this virus in French Polynesia was expected from other Pacific islands, especially from New Caledonia, because of extensive travel between the 2 French territories.

The fact that the CHIKV strain circulating in French Polynesia is closely related to the strains currently circulating in the Caribbean suggests that the French Polynesia outbreak is a result of the introduction of CHIKV from the Caribbean rather than from another Pacific island. The delay between the current outbreak and the first infected patient detected in 2014 also suggests a new introduction rather than a circulation of the strain introduced in May. However, an undetected low-level circulation of CHIKV during the cooler and drier low transmission season, simultaneously with DENV-1 circulation, cannot be excluded.

The introduction of arboviruses into French Polynesia from other French overseas territories rather than from other Pacific islands was previously reported for DENV. In 2013, DENV-3 reappeared in French Polynesia 3 months after the Solomon Islands had declared a DENV-3 outbreak. However, epidemiologic and phylogenetic investigations revealed that the DENV-3 strain that caused the outbreak in French Polynesia had been introduced by a traveler returning from French Guiana and belonged to a different genotype than the one that was circulating in the Solomon Islands (*3*).

Several conditions are favorable to a large chikungunya outbreak in French Polynesia. First, because CHIKV has never been previously reported in French Polynesia, the entire population is thought to be immunologically naive for CHIKV infection. Second, 2 potential vectors for CHIKV are present in French Polynesia: *Ae. aegypti* (*1*) and *Ae. polynesiensis* mosquitoes (*9*). Third, in French Polynesia the hot and rainy season that lasts from October through March is conducive to the proliferation of mosquitoes. We have the experience of the French Polynesian ZIKV outbreak that started with the same favorable conditions in October 2013 and was responsible for 28,000 estimated symptomatic cases from October 2013 through April 2014 (*10*). This new outbreak corroborates the recent observation that the expansion of arboviruses in the Pacific is ongoing and inevitable (*2*).
